# Assessment of depression and anxiety in adult cancer outpatients: a cross-sectional study

**DOI:** 10.1186/1471-2407-10-594

**Published:** 2010-10-29

**Authors:** Nauman A Jadoon, Waqar Munir, Mohammad A Shahzad, Zeshan S Choudhry

**Affiliations:** 1 Department of Medicine, Nishtar Medical College Hospital, Multan, Pakistan; 2 Department of Radiotherapy and Oncology, Nishtar Medical College Hospital, Multan, Pakistan

## Abstract

**Background:**

The prevalence of anxiety and depressive disorders in cancer patients and its associated factors in Pakistan is not known. There is a need to develop an evidence base to help introduce interventions as untreated depression and anxiety can lead to significant morbidity. We assessed the prevalence of depression and anxiety among adult outpatients with and without cancer as well as the effect of various demographic, clinical and behavioral factors on levels of depression and anxiety in cancer patients.

**Methods:**

This cross-sectional study was carried out in outpatient departments of Multan Institute of Nuclear Medicine and Radiotherapy and Nishtar Medical College Hospital, Multan. Aga Khan University Anxiety and Depression Scale (AKUADS) was used to define the presence of depression and anxiety in study participants. The sample consisted of 150 diagnosed cancer patients and 268 participants without cancer (control group).

**Results:**

The mean age of cancer patients was 40.85 years (SD = 16.46) and median illness duration was 5.5 months, while the mean age of the control group was 39.58 years (SD = 11.74). Overall, 66.0% of the cancer patients were found to have depression and anxiety using a cutoff score of 20 on AKUADS. Among the control group, 109 subjects (40.7%) had depression and anxiety. Cancer patients were significantly more likely to suffer from distress compared to the control group (OR = 2.83, 95% CI = 1.89-4.25, P = 0.0001). Performing logistic regression analysis showed that age up to 40 years significantly influenced the prevalence of depression and anxiety in cancer patients. There was no statistically significant difference between gender, marital status, locality, education, income, occupation, physical activity, smoking, cancer site, illness duration and mode of treatment, surgery related to cancer and presence of depression and anxiety. Cancers highly associated with depression and anxiety were gastrointestinal malignancies, chest tumors and breast cancer.

**Conclusions:**

This study highlights high prevalence rates of depression and anxiety in cancer patients. Younger age was associated with a higher likelihood of meeting criteria for psychological morbidity. The findings support screening patients for symptoms of depression and anxiety as part of standard cancer care and referring those at a higher risk of developing psychological morbidity for appropriate care.

## Background

Cancer is a serious and potentially life-threatening illness which has an effect on physical and emotional well being of patients and their families. The diagnosis of cancer is a stressful event causing significant psychological distress [1-4]. In this era of improved cancer care, it is still often believed that pain and death is inevitable for cancer patients [5].

Various studies have demonstrated high levels of depression and anxiety in cancer patients using a variety of assessment methods [1,3,4,6-8]. Depression is a challenge to study in cancer patients as symptoms occur over a range of spectrum being different in different patients [4,9]. It is a challenging job to diagnose depression in patients with cancer. It may present with guilt, worthlessness, hopelessness, lowered self esteem, social withdrawal or suicidal preoccupation [9,10]. This is further complicated by the findings that symptoms in cancer patients occur in clusters [11]. Anxiety has been shown to frequently coexist with depressive disorders [6-9]. This is significant as it has been shown that patients with comorbid anxiety and depressive disorders tend to have severe symptoms, longer recovery times, poorer outcomes and greater use of healthcare resources than those with a single disorder [12]. Prevalence and severity of psychological distress also vary across cancer types [3,4]. Anxiety and depression in cancer patients may be caused by various reasons including psychological reaction caused by diagnosis of cancer, long duration of treatment, side effects of treatment, repeated hospitalizations, disruption in life and diminished quality of life [6,13,14]. Furthermore, some of the agents act directly on central nervous system causing psychiatric morbidity [15].

Despite all these findings of higher likelihood of cancer patients to suffer from psychological distress, studies have reported that healthcare workers fail to identify cancer patients with depression and anxiety leading to under-treatment in 40-90% of the cases [10,16,17]. It is important to recognize depression in cancer patients because it may reduce chances of survival and predict early mortality [18,19]. Presence of depression and anxiety produces complications in treatment of both cancer and depression and can lead to poor compliance with treatment resulting in worsening of situation. It puts the patients at a higher risk of suicide and may produce a desire for hastened death [20]. Depression leads to a decline in patient satisfaction with medical care and predicts disease progression [21,22]. Psychological distress also has a negative effect on quality of life [23]. Depression and anxiety impair quality of life not only of the patients but also of their caregivers [24,25]. Research suggests that interventions to treat depression and anxiety are effective even in patients with advanced disease [13,26]. In addition, identification and treatment of depression and anxiety leads to reduction in disease progression improvement in survival rates, reduction in healthcare costs and improvement in quality of life [18,19,22,23,27].

There is a need to develop an evidence base to help introduce interventions as untreated depression and anxiety can lead to significant morbidity. We need prevalence estimates to be able to develop an effective strategy. Although there are a number of studies assessing psychological distress in cancer patients, there are significant gaps in literature. Majority of studies have not used a control group. This may limit generalizability of such studies due to recruitment bias. Furthermore, we have not been able to find studies investigating the association of various demographic, behavioural and clinical factors with the prevalence of depression and anxiety in cancer patients. Such studies are needed so as to identify subgroups needing intervention. Finally, there is paucity of studies on psychological distress in cancer patients from this region. There is only one study on the prevalence of depression and anxiety from Pakistan which employs a small sample size from a single centre with no control group [28]. This is the first multi-centre study from Pakistan with adequate number of patients reporting the prevalence of depression and anxiety in adult cancer outpatients. This paper outlines the results of a cross-sectional study carried out in the outpatient departments of Multan Institute of Nuclear Medicine and Radiotherapy (MINAR) and Nishtar Medical College Hospital, Multan (NMCH). The main objective of our study was to determine the prevalence of depression and anxiety in patients with cancer using a validated and reliable screening instrument and to compare them with the patients visiting medical outpatient department. We also wanted to find out the relationship of depression and anxiety with age, gender, locality, education, income and occupation, type of cancer, current treatment modality, duration of illness and history of surgical intervention related to the disease, smoking and physical activity.

## Methods

A multi-centre, cross-sectional study was carried out in the outpatient departments of MINAR and NMCH, Multan to determine the prevalence of depression and anxiety in adult cancer outpatients. Consecutive patients visiting outpatient department were recruited for the study. Patients were included if they had a cancer diagnosis, were in the age range 18-70 years, consented to participate in the study, and were able to complete questionnaire. Exclusion criteria were diagnosis at current visit, critical condition, language or hearing problem, cognitive impairment, incomplete records or ongoing psychological treatment. The authors approached all the eligible patients and requested their consent to participate in the study after explaining the study. Controls were randomly selected from different outpatient departments of the same centres to reduce potential differences in socioeconomic and educational factors. Every fifth patient visiting the medical outpatient department was recruited in the study after taking informed consent.

Anxiety and depression were assessed using a self administered structured questionnaire. The questionnaire was administered after taking written informed consent from the participants. Data on basic demographic details including age, gender, locality, education, income and occupation were collected. Cancer related variables including type of cancer, current treatment modality, duration of illness and history of surgical intervention related to the disease were obtained from the medical record of the patients. Behavioral factors included in the questionnaire to determine their association with depression and anxiety were smoking and physical activity which were self reported. Patients were labelled smokers if they had smoked for more than one year. Physical activity was defined as exercise for more than 3 hours per week. Anxiety and depression was assessed using Aga Khan University Anxiety and Depression Scale (AKUADS). It is a 25-item self administered instrument that measures the presence of symptomatology of anxiety and depression in adults. It is a widely used instrument developed indigenously in Urdu. Its validation study has shown that it is a reliable and valid instrument to assess psychological morbidity in patients. It has 13 psychological and 12 somatic items which measure presence and severity of symptoms making it superior to other available instruments in Urdu containing only either psychological or somatic items [29,30]. At a cut-off score of 20, it has a sensitivity of 66%, a specificity of 79%, a positive predictive value of 83 and a negative predictive value of 60. It has a good level of reliability and analysis showed item-item correlation ≥ 0.75 [29,30]. The data from the questionnaire was entered as categorical data on the basis of cutoff for analysis.

Data was analyzed using Statistical Package for Social Sciences 16 (SPSS Inc., Chicago, IL, USA). Descriptive analyses were recorded as means and proportions. Logistic regression analysis was employed to determine the association of various demographic, clinical and behavioral factors with the presence of depression and anxiety in cancer patients. A p value of < 0.05 was considered statistically significant.

The study was approved by ethics review committee of NMCH, Multan. Written informed consent was obtained from all the participants accrued in the study.

## Results

Out of a total of 200 cancer patients selected, 186 were determined to be eligible for survey. Fourteen patients were ineligible on account of diagnosis on recent visit (4), critical condition (1), cognitive impairment (4), language problem (2) or ongoing psychiatric treatment (3). With a response rate of 90.9%, 169 patients consented to participate in the study and completed questionnaires. After excluding incompletely filled questionnaires, 150 participants were included in final analysis. The mean age of cancer patients was 40.85 years (SD = 16.46 years) and median duration of illness was 5.50 months. Majority of the patients were male (64.0%), married (79.3%), from urban locality (53.3%), illiterate (44.0%), belonging to low socioeconomic group (66.7%), and unemployed (29.3%). Most of the patients were non-smokers (71.3%) and physically active (56.0%). The most common diagnoses were urological (21.3%), hematological (18.0%), and head and neck malignancies (15.0%) and breast (14.0%) cancer. A control group of 300 individuals was selected out of which 293 were eligible for accrual. In total, 281 subjects consented and filled the study questionnaire with a response rate of 95.9%. Control subjects included in the final analysis were 268 who had completely filled out questionnaires. The mean age of control group was 39.58 years (SD = 11.74 years). Demographics and clinical characteristics of cancer subjects are presented in table 1 along with demographics of control group. Since the control group accrual was done from waiting rooms of medical outpatient department, data about their diagnoses is not available.

**Table 1:** Participant Characteristics

Variables	Cancer Subjects No. (%)	Control Subjects No. (%)
**Age**		

≤ 40 Years	78(52.0)	166(61.9)

> 40 Years	72(48.0)	102(38.1)

**Gender**		

Male	96(64.0)	158(59.0)

Female	54(36.0)	110(41.0)

**Marital Status**		

Married	119(79.3)	193(72.0)

Unmarried	31(20.7)	75(28.0)

**Locality**		

Urban	80(53.3)	139(51.9)

Rural	70(46.7)	129(48.1)

**Education**		

Illiterate	60(40.0)	80(29.9)

Primary	19(12.7)	42(15.7)

Secondary	31(20.7)	68(25.4)

High School Certificate	19(12.7)	40(14.9)

Bachelor	17(11.3)	23(8.6)

Masters	4(2.7)	15(5.6)

**Income**		

< Rs. 10,000	100(66.7)	147(54.9)

Rs.10,000-Rs.20,000	41(27.3)	50(18.7)

Rs.20,000-Rs.40,000	6(4.0)	43(16.0)

> Rs. 40,000	3(2.0)	28(10.4)

**Occupation**		

Professional	13(8.7)	20(7.5)

Skilled worker	10(6.7)	29(10.8)

Administrative Job	11(7.3)	23(8.6)

Sales	19(12.7)	55(20.5)

Farming	24(16.0)	12(4.5)

Unskilled/Elementary	29(19.3)	31(11.6)

Unemployed	44(29.3)	98(36.6)

**Physical Activity**		

Active	84(56.0)	

Inactive	66(44.0)	

**Smoking**		

Smoker	43(28.7)	

Non-Smoker	107(71.3)	

**Cancer**		

Hematological	27(18.0)	

Head & Neck	23(15.3)	

Urological	32(21.3)	

Breast	21(14.0)	

Chest	13(8.7)	

Sarcoma	11(7.3)	

Squamous & Basal Cell	1(0.7)	

Brain	7(4.7)	

GIT	15(10.0)	

**Duration**		

≤ 6 Months	88(58.7)	

> 6 Months	62(41.3)	

**Treatment**		

Chemotherapy	55(36.7)	

Radiotherapy	22(14.7)	

Concomitant	59(39.3)	

None	14(9.3)	

**Surgery**		

Yes	63(42.0)	

No	87(58.0)	

Overall, 66.0% of the cancer patients were found to have depression and anxiety using a cut-off score of 20 on AKUADS. Among the control group, 109 subjects (40.7%) had depression and anxiety. This difference in the prevalence rates was found to be statistically significant (p < 0.001). Cancer patients were 2.83 times more likely to have psychological distress (95% CI = 1.89-4.25). Cancers with the highest prevalence of depression and anxiety were gastrointestinal malignancies, chest tumors and breast cancer.

Table 2 describes the results of logistic regression analysis performed for determining the association of various demographic, clinical and behavioral factors with the presence of depression and anxiety in cancer patients. Age less than 40 years was found to significantly increase the odds of having depression and anxiety among cancer patients. The findings showed that among different demographic variables, there were no significant differences among anxiety, depression and patients' gender, marital status, locality, educational background, socioeconomic status, and occupation. The results also indicated that type of malignancy, duration of illness, type of intervention and surgery related to disease did not significantly alter the odds of having depression and anxiety. Finally among behavioral factors, both smoking and physical activity did not significantly alter the odds of suffering from depression and anxiety.

**Table 2:** The Results of Logistic Regression Analysis showing association of variables with Psychological distress in cancer patients

Variables	OR	95% CI	P
**Age**			0.027
	
≤ 40 Years	1		
	
> 40 Years	0.46	0.23-0.91	

**Gender**			0.900
	
Male	1		
	
Female	1.05	0.52-2.12	

**Marital Status**			0.297
	
Married	1		
	
Unmarried	0.65	0.29-1.46	

**Locality**			0.534
	
Urban	1		
	
Rural	1.24	0.63-2.45	

**Education**			0.727
	
Illiterate	1		
	
Primary	0.5	0.07-3.81	
	
Secondary	0.27	0.03-2.53	
	
High School Certificate	0.63	0.08-5.10	
	
Bachelors	0.73	0.08-6.31	
	
Masters		0.05-3.84	

**Income**			0.641
	
< Rs. 10,000	1		
	
Rs.10,000-Rs.20,000	0.27	0.02-3.08	
	
Rs.20,000-Rs.40,000	0.21	0.02-2.5	
	
> Rs. 40,000	0.25	0.01-4.73	

**Occupation**			0.714
	
Professional	1		
	
Skilled worker	0.48	0.12-1.98	
	
Administrative Job	0.68	0.15-2.99	
	
Sales	1.91	0.50-7.23	
	
Farming	0.57	0.17-1.86	
	
Unskilled/Elementary	0.79	0.28-2.25	
	
Unemployed	0.72	0.27-1.93	

**Physical Activity**			0.397
	
Active	1		
	
Inactive	1.35	0.68-2.67	

**Smoking**			0.082
	
Smoker	1	0.747	
	
Non-Smoker	0.49	0.22-1.00	

**Cancer**			0.144
	
Hematological	1		
	
Head & Neck	7.0	1.32-37.15	
	
Urological	3.47	0.62-19.33	
	
Breast	3.9	0.75-20.34	
	
Chest	2.03	0.34-12.24	
	
Sarcoma	5.42	0.04-60.77	
	
Squamous & Basal Cell	5.41	0.81-36.36	
	
Brain	1.24	0.23-6.62	
	
GIT	8.67	1.05-71.57	

**Duration**			0.747
	
≤ 6 Months	1		
	
> 6 Months	1.12	0.56-2.22	

**Treatment**			0.995
	
Chemotherapy	1		
	
Radiotherapy	0.95	0.28-3.24	
	
Concomitant	0.84	0.20-3.46	
	
None	0.92	0.27-3.12	

**Surgery**			0.234
	
Yes	1		
	
No	0.66	0.33-1.32	

## Discussion

The main finding of this study is the high prevalence of depression and anxiety in cancer patients as compared to the control group (66.0% vs. 40.7%). In our study, 66% patients with cancer had depression and anxiety. This is slightly higher than some studies conducted earlier which reported prevalence of depression spectrum syndromes ranging from 0-58% [1,3,4]. However, the findings are comparable to a recent study from Pakistan reporting prevalence rates of 52% in cancer patients and a study from Iran showing rates of 47.2% and 57% for depression and anxiety respectively [28,31]. There can be a number of reasons for this difference in prevalence rates. A first explanation could be that most of the studies on prevalence of psychological morbidity in cancer patients are from developed countries which have low prevalence of mental health problems as compared to developing countries [32]. A second explanation could be that prevalence of psychological morbidity even in general population of Pakistan is much higher, as documented by a systematic review reporting prevalence rate of 34% in general population which is close to the level of psychological morbidity in control group in our study [33]. A third explanation could be that many of the participants in our study were from rural areas, illiterate, and had a low household income which is established risk factors for psychiatric morbidity [28]. Also the prevailing situation in the country puts them at even higher risk as country is facing socio-political instability, economic crises and growing unemployment.

This is the first study to our knowledge from Pakistan reporting depression and anxiety in cancer patients from two centres with a control group using a valid and reliable instrument. In our study, a significant association was observed between psychological morbidity and age with patients of age up to 40 years having a higher likelihood of suffering from depression and anxiety. This is in agreement with previous studies demonstrating that younger people are more prone to psychological distress when suffering from cancer [31,34,35].

There was no statistically significant relationship between depression, anxiety and various demographic factors in the present study. A meta-analysis of 58 studies showed that there were significant differences among groups with regards to sex, age and type of cancer [36]. We, however, did not find any significant effect of gender on predisposition to psychological morbidity. Marital status, locality, education, income, and occupation also did not affect psychological morbidity presence in our study. In addition, there was no significant association of depression and anxiety with cancer type, duration, treatment, or surgery related to disease. A study from Japan, however, reported increased levels of depression in cancer patients undergoing surgery [37].

Strengths of our study include standardized measures, use of control group, analysis of factors that may affect prevalence of depression and anxiety and a good response rate. The goal of unbiased control was achieved by recruiting both cases and controls at the same time and under same conditions. Furthermore, both the cases and controls were from the same source population and were evaluated using the same instrument. We used a valid and reliable questionnaire developed indigenously in Urdu to reduce the potential of error caused by instruments developed from populations belonging to different cultural and socioeconomic setups. The study also has certain limitations including use of self reported questionnaires instead of diagnostic interview and unavailability of diagnosis for the control group.

In order to provide optimum care for cancer patients incorporating management of physical as well as psychological symptoms, it is necessary to increase awareness about prevalence of psychological morbidity among cancer patients and improve identification of symptoms [9]. It is recommended that all the cancer patients should complete a psychological screening on baseline as a part of standard cancer care. The oncologist should then refer the patients for appropriate care on the basis of results of screening. The concern expressed by some clinicians of bothering patients is unfounded as most of the patients are thankful for the opportunity to answer questions about their feelings [38]. It has been demonstrated that even a short psychological intervention delivered by nonspecialist is effective and promotes adjustment among cancer patients at a high risk of developing depression and anxiety [26,39]. This has also been recommended by NCCN clinical practice guidelines which recommend screening of patients for distress and psychotherapy subsequently for those with moderate to severe distress [2,40].

## Conclusions

The study shows that compared with control group, adults with cancer have higher prevalence rates of depression and anxiety. Younger age was associated with a higher likelihood of meeting criteria for psychological morbidity. The findings support screening patients for symptoms of depression and anxiety as part of standard cancer care and referring those at a higher risk of developing psychological morbidity for appropriate care. This will lead to decreased morbidity, improvement in disease outcome, reduction in healthcare costs, and improvement in quality of life of patients.

## List of Abbreviations

SCID: Structured Clinical Interview for DSM Disorders; NCCN: National Comprehensive Cancer Network; Rs: Rupees

## Competing interests

The authors declare that they have no competing interests.

## Authors' contributions

NAJ conceived and designed the study, participated in data acquisition, analyzed the data and drafted the manuscript. WM participated in data collection, analysis of data and reviewing of manuscript. MAS collected data and helped in data analysis and revision of final draft of manuscript. ZSC contributed to data collection and analysis. All authors read and approved the final manuscript.

## Pre-publication history

The pre-publication history for this paper can be accessed here:

http://www.biomedcentral.com/1471-2407/10/594/prepub
